# Shear stress augments mitochondrial ATP generation that triggers ATP release and Ca^2+^ signaling in vascular endothelial cells

**DOI:** 10.1152/ajpheart.00204.2018

**Published:** 2018-08-24

**Authors:** Kimiko Yamamoto, Hiromi Imamura, Joji Ando

**Affiliations:** ^1^Laboratory of System Physiology, Department of Biomedical Engineering, Graduate School of Medicine, University of Tokyo, Tokyo, Japan; ^2^Laboratory of Functional Biology, Graduate School of Biostudies, Kyoto University, Kyoto, Japan; ^3^Laboratory of Biomedical Engineering, School of Medicine, Dokkyo Medical University, Tochigi, Japan

**Keywords:** ATP, calcium signaling, endothelial cells, mitochondria, shear stress

## Abstract

Vascular endothelial cells (ECs) sense and transduce hemodynamic shear stress into intracellular biochemical signals, and Ca^2+^ signaling plays a critical role in this mechanotransduction, i.e., ECs release ATP in the caveolae in response to shear stress and, in turn, the released ATP activates P2 purinoceptors, which results in an influx into the cells of extracellular Ca^2+^. However, the mechanism by which the shear stress evokes ATP release remains unclear. Here, we demonstrated that cellular mitochondria play a critical role in this process. Cultured human pulmonary artery ECs were exposed to controlled levels of shear stress in a flow-loading device, and changes in the mitochondrial ATP levels were examined by real-time imaging using a fluorescence resonance energy transfer-based ATP biosensor. Immediately upon exposure of the cells to flow, mitochondrial ATP levels increased, which was both reversible and dependent on the intensity of shear stress. Inhibitors of the mitochondrial electron transport chain and ATP synthase as well as knockdown of caveolin-1, a major structural protein of the caveolae, abolished the shear stress-induced mitochondrial ATP generation, resulting in the loss of ATP release and influx of Ca^2+^ into the cells. These results suggest the novel role of mitochondria in transducing shear stress into ATP generation: ATP generation leads to ATP release in the caveolae, triggering purinergic Ca^2+^ signaling. Thus, exposure of ECs to shear stress seems to activate mitochondrial ATP generation through caveola- or caveolin-1-mediated mechanisms.

**NEW & NOTEWORTHY** The mechanism of how vascular endothelial cells sense shear stress generated by blood flow and transduce it into functional responses remains unclear. Real-time imaging of mitochondrial ATP demonstrated the novel role of endothelial mitochondria as mechanosignaling organelles that are able to transduce shear stress into ATP generation, triggering ATP release and purinoceptor-mediated Ca^2+^ signaling within the cells.

## INTRODUCTION

The endothelial cells (ECs) that line the lumens of blood vessels are constantly exposed to a fluid dynamic force, the shear stress generated by the flow of blood. ECs sense this shear stress and transduce it into intracellular biochemical signals, resulting in changes in the morphology, functions, and gene expression profiles of cells (13). These EC mechanoresponses are critical for the homeostasis of the circulatory system, and their impairment can lead to the development of various vascular diseases, including hypertension, aneurysm, thrombosis, and atherosclerosis (5, 11, 40).

A large number of studies have focused on clarifying the mechanisms of EC mechanotransduction. A unique feature found to be associated with exposure of the cells to shear stress is the almost simultaneous activation of multiple intracellular signal transduction pathways through various membrane molecules and microdomains (4). Ca^2+^ signaling mediated by purinergic receptors has been shown to serve as one of these shear stress mechanotransduction pathways (3, 35). When exposed to shear stress, ECs rapidly release intrinsic ATP; this, in turn, activates P2X4 and P2Y2 receptors, triggering the influx of extracellular Ca^2+^ and Ca^2+^ release from intracellular Ca^2+^ stores. The resulting increase in the cytoplasmic Ca^2+^ concentration results in the augmented production of nitric oxide, which plays a crucial role in the regulation of blood pressure, blood flow-dependent vasodilation, and vascular remodeling in tissues (40).

In recent years, we have developed a novel chemiluminescence method for imaging cell surface ATP. This has demonstrated that shear stress causes a focal release of ATP in the caveolae-rich regions of EC plasma membranes, with concentrations exceeding 10 μM (37). Coimaging of ATP and Ca^2+^ showed that the Ca^2+^ influx started at the site of focal ATP release, with the increase in cytoplasmic Ca^2+^concentration propagating through the entire cell as a Ca^2+^ wave. However, it remains unclear how exposure of the cells to shear stress evokes the release of ATP.

In the present study, we investigated possible roles of mitochondria in ATP release. We used a fluorescence resonance energy transfer (FRET)-based ATP biosensor (20) to visualize the ATP inside the mitochondria of cultured human pulmonary aortic ECs (HPAECs) and analyzed the changes in mitochondrial ATP generation after exposure to shear stress. We also examined how membrane microdomain caveolae (2) are involved in mitochondrial responses to shear stress.

## MATERIALS AND METHODS

### 

#### Cell culture.

HPAECs were purchased from Lonza Walkersville (product code: CC-2530) and grown in medium 199 supplemented with 15% FBS, 2 mM l-glutamine (GIBCO), 50 µg/ml heparin, and 30 µg/ml EC growth factor (Becton Dickinson) in a 1% gelatin-coated tissue culture flask. Cells used in the experiments in this study were from *passages 7−10*. All experiments were approved by the Ethics Committee of The University of Tokyo Graduate School of Medicine.

#### Shear stress experiments.

Cells were subjected to controlled levels of laminar shear stress in a parallel plate-type flow-loading device (FCS2, Bioptechs), as previously described (41). Briefly, the flow chamber consisted of a 1% gelatin-coated cover glass with the cultured cells on one side and a second parallel glass plate held 200 μm apart from the first by a Teflon gasket. The medium was perfused with a roller/tube pump, and the entire closed circuit was maintained at exactly 37°C. The intensity of the shear stress (τ; in dyn/cm^2^) acting on the EC layer was calculated from the following formula: τ = 6μQ/*a*^2^*b*, where μ is the viscosity of the perfusate (in Poise), Q is the flow volume (in ml/s), and *a* and *b* are the cross-sectional dimensions of the flow path (in cm).

#### Imaging of mitochondrial ATP.

Mitochondrial ATP was imaged using a FRET-based ATP indicator based on the ε-subunit for the analytic measurement biosensor targeted at the mitochondrial matrix (mitAT1.03). cDNA of mitAT1.03 was ligated and cloned into the SalI/NotI sites of a pAd-CMV-V5-DEST Gateway vector (ThermoFisher Scientific), and the adenovirus vector containing mitAT1.03 cDNA (Ad-mitAT1.03) was constructed as previously described (20). Cultured HPAECs were infected with 50 plaque-forming units/cell of Ad-mitAT1.03.

Three to five days after Ad-mitAT1.03 infection, HPAECs (maintained at 37°C) were imaged on an ECLIPSE Ti-E inverted microscope (Nikon) with ×60 and ×100 Apo total internal reflection fluorescence oil objectives (1.49 numerical aperture) using a water-cooling electron multiplier charge-coupled device camera (ImagEM C9100-13, Hamamatsu) controlled by HCImage software (version 4.3, Hamamatsu). The dual-emission ratio imaging of mitAT1.03 used a FF01-427/10 excitation filter (Semrock), FF458-Di01 dichroic mirror, and Dual View Multichannel Imaging System (DV2, Photometrics) equipped with two emission filters [FF01-483/32 for cyan fluorescent protein (CFP) and FF01-542/27 for yellow fluorescent protein (YFP)]. Images were analyzed using the MetaMorph software program (version 7.7, Molecular Devices).

#### Characterization of AT1.03 protein.

The ATP sensitivity of the ATP indicator based on the ε-subunit for analytic measurements (AT1.03) was examined in vitro. The concentration of purified AT1.03 protein was adjusted with buffer [containing 50 mM MOPS-KOH (pH 7.3), 50 mM KCl, 0.5 mM MgCl_2_, and 0.05% Triton X-100] so that the fluorescent intensity of CFP was equivalent to that observed in HPAECs using the same microscope system. Changes in CFP and YFP emissions for a different concentration of ATP magnesium salt were measured at 37°C, and YFP-to-CFP emission ratios were calculated.

#### Measurement of extracellular ATP.

ATP released by HPAECs was measured by means of a luciferin-luciferase assay, as previously described (41). Cells were exposed to laminar flow in a flow chamber. The perfusate flowing out of the chamber was collected every 10 s, and 100 µl were applied to the ATP assay system (Toyo Ink, Tokyo). The ATP assay mixture (luciferase, D-luciferin, and BSA) was injected into each sample, and the relative light intensity was recorded for 10 s in a Wallac 1420 ARVO SX multilabel counter (Perkin-Elmer). A calibration curve for ATP concentrations was obtained for each experiment using the same batch of luciferin-luciferase reagents.

#### Immunohistochemistry.

Cells were fixed with 4% paraformaldehyde (Sigma) and maintained in 1% normal BSA (Sigma) to block nonspecific protein binding sites. Cells were incubated with rabbit anticaveolin-1 polyclonal antibody (BD Transduction Laboratories); after a wash, they were incubated with Alexa Fluor 488 goat anti-rabbit IgG (ThermoFisher Scientific) at 1:500 dilution. Stained cells were photographed through a confocal fluorescence microscope (Leica), and images were imported into Adobe Photoshop as TIFFs for figure assembly.

#### Ca^2+^ measurements.

Cells were loaded with the Ca^2+^-sensitive dye Fluo-4-AM (5 µM, Dojindo) and placed in the FCS2 flow chamber (Bioptechs) on the stage of an ECLIPSE Ti-E inverted microscope (Nikon). Fluo-4 was excited with light that passed through a 490-nm bandpass filter (Lambda DG-4), and the emitted light was guided through a 510-nm bandpass filter to a water-cooling electron multiplier charge-coupled device camera (ImagEM C9100-13, Hamamatsu). Fluorescence intensity is a reflection of the intracellular Ca^2+^ concentration ([Ca^2+^]_i_). Ca^2+^ images were acquired sequentially as full-frame images (512 × 512 pixels) with an exposure period of 30 ms. Images were analyzed using HCImage software (version 4.3, Hamamatsu).

#### siRNA preparation and transfection.

siRNA was used to knock down caveolin-1 expression in HPAECs, as previously described (37). A DNA fragment flanked by the BamHI and Hind III sites was synthesized; this contained the sense target sequence corresponding to bases 167–185 from the open reading frame of human caveolin-1 (GenBank Accession No: NM_001753, 5′-CTAAACACCTCAACGATGA-3′), the hairpin loop sequence (5′-CTGTGAAGCCACAGATGGG-3′), and the antisense target sequence (5′-TCATCGTTGAGGTGTTTAG-3′). The fragment was inserted between the human U6 promoter and the terminator sequences of pBAsi-hU6 (Takara) to generate a stem-loop type of siRNA in transfected cells. The randomized sequence 5′-TAACATGAACCACGACTAC-3′ was used to construct the vector for a negative control (scrambled siRNA). HPAECs were treated with the construct using Lipofectamine 2000 (Invitrogen) as the transfection reagent. Experiments were conducted 48 h after transfection.

#### Statistical analysis.

Results are presented as means ± SD. Statistical significance was evaluated by ANOVA with the Bonferonni adjustment applied to the results of post hoc *t*-tests. Statistical analysis was performed with SPSS software (SPSS). *P* values of <0.05 were regarded as being statistically significant.

## RESULTS

### 

#### Shear stress resulted in a rapid augmentation of mitochondrial ATP generation.

**Fig. 1.**
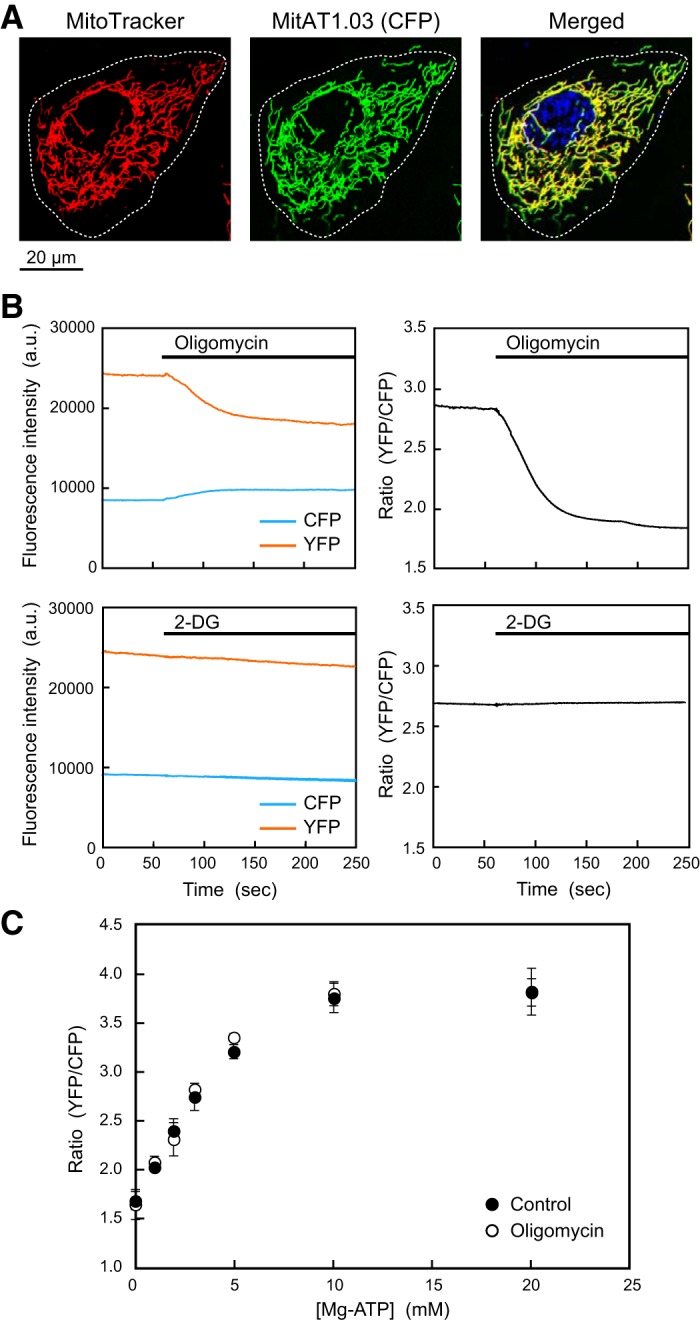
Monitoring of mitochondrial ATP levels in human pulmonary artery endothelial cells. *A*: distribution of the fluorescence resonance energy transfer-based ATP biosensor mitAT1.03. This was correctly located in mitochondria stained with MitoTracker deep red FM. Cell nuclei were stained with Hoechst 33342, and the cell margin obtained visualized under a phase-contrast microscope is shown by the dashed lines. *B*: time course of the fluorescence intensity of cyan fluorescent protein (CFP; blue) and yellow fluorescent protein (YFP; red) inside the regions of interest. Treatment of cells with the ATP synthase inhibitor oligomycin (10 µg/ml) induced a decrease in YFP intensity and an increase in CFP intensity. The YFP-to-CFP ratio therefore decreased, indicating a decrease in ATP levels. Treatment of cells with an inhibitor of glycolysis [2-deoxy-d-glucose (2-DG); 10 mM] had no effect on the YFP-to-CFP ratio. *C*: ATP sensitivity of purified AT1.03. The plot of YFP-to-CFP ratios against Mg-ATP concentrations showed that the YFP-to-CFP ratio increased in an ATP concentration-dependent manner in the range of 0–10 mM. Oligomycin (10 µg/ml) had no effect on the ATP sensitivity of AT1.03. a.u., Arbitrary units.

To monitor changes in mitochondrial ATP levels, we used a genetically encoded FRET-based ATP biosensor that targeted mitochondrial matrix mitAT1.03. The localization of mitAT1.03 coincided precisely with the mitochondria, as shown by live-cell imaging using the mitochondrion-selective dye MitoTracker (Fig. 1*A*). When ECs were treated with oligomycin, an inhibitor of ATP synthase, the fluorescence of mitAT1.03 YFP decreased and fluorescence of CFP increased, thereby reducing the YFP-to-CFP ratio; this indicated a decrease in mitochondrial ATP generation (Fig. 1*B*). In contrast, the fluorescence of mitAT1.03 was not affected by treatment of cells with 2-deoxy-d-glucose (2-DG), an inhibitor of cytosolic glycolysis. An in vitro examination of the ATP sensitivity of purified AT1.03 showed a clear correlation between the YFP-to-CFP ratio and ATP concentration in the range of 0–10 mM (Fig. 1*C*). Oligomycin had no effect on the ATP sensitivity of purified AT1.03. These findings indicated that AT1.03 enabled real-time and quantitative observation of mitochondrial ATP levels.

**Fig. 2.**
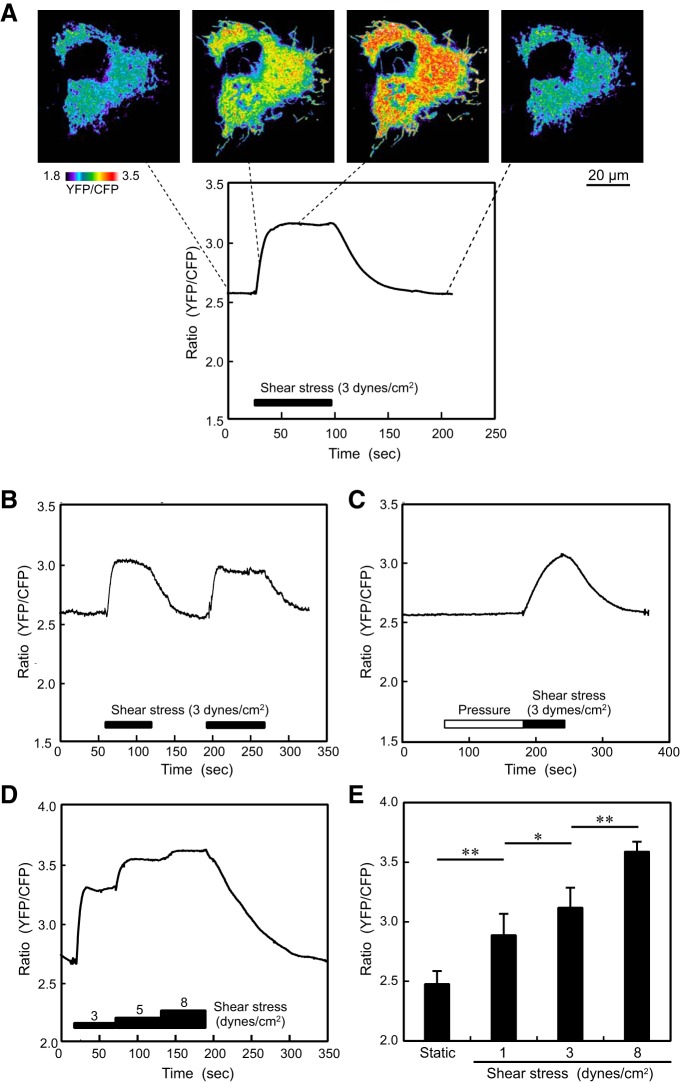
Effects of shear stress on mitochondrial ATP levels. *A*: pseudocolor images of the yellow fluorescent protein (YFP)-to-cyan fluorescent protein (CFP) ratio before, during, and after the application of shear stress. The colors represent the ATP levels indicated by the scale. Shear stress (3 dyn/cm^2^) increased ATP levels over the entire mitochondria. The time course of the YFP-to-CFP ratio showed that the ATP level rapidly increased in response to shear stress, remained at the increased level, and then returned to the control level after the shear stress ceased. *B*: response to the repeated application of shear stress. There was an increase in mitochondrial ATP levels in response to each application of shear stress. Similar findings were observed in many cells. *C*: effects of hydrostatic pressure on mitochondrial ATP levels. Hydrostatic pressure (40 mmHg) had no effect on ATP levels. In contrast, there was a marked increase in ATP levels in response to shear stress. *D*: intensity dependency of the shear stress-induced changes in mitochondrial ATP levels. ATP levels in the mitochondria increased further as the intensity of shear stress increased. *E*: bar graph showing the results of a quantitative analysis of the shear stress-induced changes in ATP levels. Values are means ± SD of the data obtained in 15 cells. *P < 0.05; **P < 0.01.

HPAECs were exposed to controlled levels of shear stress in a flow-loading apparatus, and changes in mitochondrial ATP levels were examined. Pseudocolor images of the YFP-to-CFP ratio showed increased ATP levels over the entire mitochondria when cells were exposed to shear stress (Fig. 2*A*). A rough estimate using the correlation curve between the YFP-to-CFP ratio and ATP concentration suggested that ATP levels had approximately doubled. Changes in ATP levels over time were quantified by defining regions of interest in the mitochondria. The ATP level increased immediately after exposure of the cells to shear stress (see Supplemental Video S1 in the Supplemental Material available at the *American Journal of Physiology-Heart and Circulatory Physiology* website); this level was maintained throughout the exposure to shear stress and then decreased after the shear stress ceased.

#### Mitochondrial ATP responses to shear stress were reversible and dose dependent.

When shear stress was applied repeatedly, ATP levels in the mitochondria increased repeatedly in response, indicating that the mitochondrial response was reversible (Fig. 2*B*). When ECs were exposed to shear stresses at different intensities from 3 to 8 dyn/cm^2^, ATP levels increased in a dose-dependent manner (Fig. 2, *D* and *E*). In the flow-loading experiments, ECs were subjected not only to shear stress but also to pressure that exerts a compressive force on the cells. To examine whether pressure affected mitochondrial ATP levels, we exposed ECs to hydrostatic pressure under a no-flow condition. Hydrostatic pressure alone had no effect on ATP levels, but when the same cells were exposed to flow in addition to pressure, ATP levels clearly increased (Fig. 2*C*). These findings indicated that the flow-induced increase in mitochondrial ATP generation was attributable to shear stress and not the pressure to which cells were exposed.

#### Shear stress activated mitochondrial oxidative phosphorylation.

**Fig. 3.**
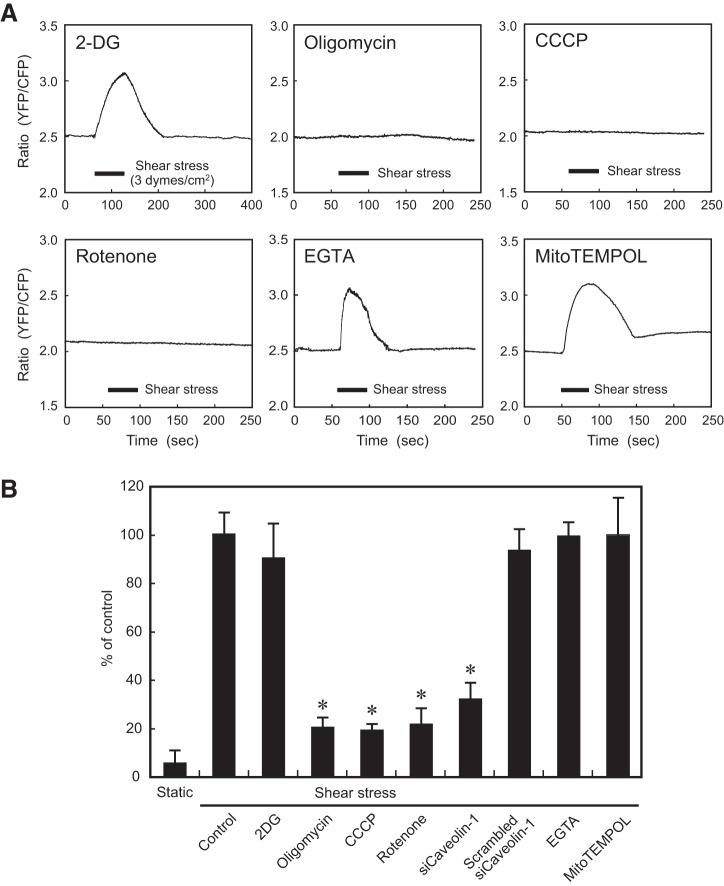
Effects of inhibitors of glycolysis and mitochondrial oxidative phosphorylation on the shear stress-induced increase in ATP generation. *A*: temporal changes in mitochondrial ATP levels. Shear stress induced an increase in ATP levels in cells treated with the glycolysis inhibitor 2-deoxy-d-gllucose (2-DG; 10 mM) but not in cells treated with an ATP synthase inhibitor (oligomycin, 10 µg/ml), a mitochondrial oxidative phosphorylation uncoupler [carbonyl cyanide *m*-chlorophenylhydrazone (CCCP); 0.4 µg/ml], and a mitochondrial electron transport inhibitor (rotenone, 5 µM). This indicated that shear stress activated the function of mitochondrial oxidative phosphorylation. Neither the addition of EGTA (1 mM) into the culture medium, which chelates extracellular Ca^2+^, nor treatment of endothelial cells (ECs) with MitoTEMPOL (50 μM), which inactivates mitochondrial ROS, had an effect on ATP generation. Baseline mitochondrial ATP concentrations were reduced by treatment of ECs with oligomycin, CCCP, or rotenone, whereas no such change was observed after treatment of cells with 2-DG, EGTA, or MitoTEMPOL. These mitochondrial ATP responses were representative of those of dozens of cells, all of which showed similar results. *B*: extracellular ATP release in response to shear stress. Human pulmonary artery ECs (HPAECs) were exposed to shear stress (3 dyn/cm^2^) for 1 min, and the effluent was subjected to ATP measurement. HPAECs released ATP in response to shear stress, and inhibitors of the mitochondrial oxidative phosphorylation (oligomycin, CCCP, and rotenone) markedly suppressed ATP release, whereas glycolysis inhibitor (2-DG), EGTA, and MitoTEMPOL had no effect. Knockdown of caveolin-1 with its siRNA significantly suppressed ATP release, whereas scrambled siRNA had no effect. Results are presented as means ± SD of nine samples obtained in three separate experiments. *P < 0.01 compared with control.

Before exposure to shear stress, ECs were treated with one of three inhibitors: oligomycin (an ATP synthase inhibitor), carbonyl cyanide *m*-chlorophenylhydrazone (CCCP; a mitochondrial oxidative phosphorylation uncoupler), or rotenone (an inhibitor of mitochondrial electron transport). All three inhibitors abolished shear stress-induced mitochondrial ATP generation in ECs (Fig. 3*A*). In contrast, treatment of cells with the glycolysis inhibitor 2-DG had no such effect. These findings indicate that exposure of ECs to shear stress increased mitochondrial ATP generation by activating mitochondrial oxidative phosphorylation. Neither addition of EGTA to culture media, which removes extracellular Ca^2+^, nor treatment of ECs with MitoTEMPOL, which inactivates mitochondrial reactive oxygen species (ROS), exerted any influence on the ATP generation. These findings indicate that the influx of extracellular Ca^2+^ and ROS produced by mitochondria do not play a part in shear stress-induced mitochondrial ATP generation.

#### Mitochondria were the major source of ATP released by ECs in response to shear stress.

HPAECs were exposed to shear stress, and changes in ATP concentration were measured biochemically in a portion of the perfusate, as described above in materials and methods. Exposure of ECs to shear stress significantly increased the amount of ATP released from cells (Fig. 3*B*), which was in good agreement with data obtained in our previous study (41). Shear stress-induced ATP release was suppressed markedly by treatment of ECs with oligomycin, CCCP, or rotenone, but treatment of cells with 2-DG, EGTA, and MitoTEMPOL had no effect on ATP release. These findings indicated that mitochondrial ATP generation plays a crucial role in shear stress-induced ATP release from cells.

#### Caveolae were implicated in shear stress-induced mitochondrial ATP generation.

**Fig. 4.**
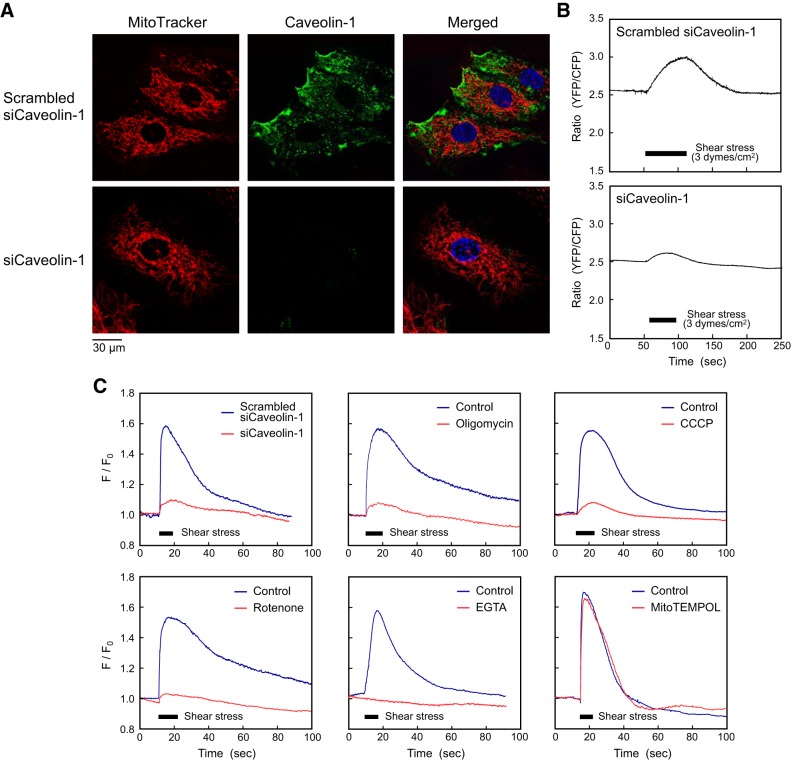
Involvement of caveolin-1 in shear stress-induced ATP generation and Ca^2+^ signaling. *A*: relationship between mitochondria and caveolin-1, a marker protein for caveolae. After mitochondria were imaged with MitoTracker deep red FM, human pulmonary artery endothelial cells (HPAECs) were immunostained with antibody to caveolin-1. Cell nuclei were stained with DAPI. Caveolin-1 was unevenly distributed over the cell surface and was concentrated at a specific part of the cell periphery. The edge of the mitochondria was in close proximity to the region with concentrated caveolin-1. Caveolin-1 was completely knocked down by its siRNA. *B*: temporal changes in mitochondrial ATP levels under shear stress. Shear stress increased mitochondrial ATP levels in cells treated with caveolin-1-scrambled siRNA, whereas caveolin-1 knockdown by its siRNA abolished shear stress-induced mitochondrial ATP generation. These mitochondrial ATP responses were representative of those of dozens of cells, all of which showed similar results. *C*: Ca^2+^ responses to shear stress in HPAECs. Intracellular Ca^2+^ concentrations ([Ca^2+^]_i_) are expressed as the ratio of change in Fluo-4 fluorescence to the control before application of shear stress (F/F_0_). Closed rectangles indicate the duration of shear stress (3 dyn/cm^2^). Shear stress induced a marked increase in [Ca^2+^]_i_ in cells treated with caveolin-1-scrambled siRNA but not in cells treated with caveolin-1 siRNA. Treatment of cells with oligomycin (10 µg/ml), carbonyl cyanide *m*-chlorophenylhydrazone (CCCP; 0.4 µg/ml), or rotenone (5 µM) abolished the shear stress-mediated Ca^2+^ response. The Ca^2+^ response was blocked by EGTA (1 mM) but not by MitoTEMPOL (50 μM). No changes in baseline [Ca^2+^]_i_ were observed after treatment of cells with any of the above inhibitors or siRNA. Treatment of cells with these Ca^2+^response curves are representative of those of dozens of cells, all of which showed similar results. These results indicate that caveolin-1 and mitochondrial ATP generation can be implicated in the Ca^2+^ signaling after shear stress. Scrambled siCaveolin-1, cells transfected with caveolin-1-scrambled siRNA; siCaveolin-1, cells transfected with caveolin-1 siRNA; CFP, cyan fluorescent protein; YFP, yellow fluorescent protein.

HPAECs are rich in caveolae, which are small flask-shaped invaginations of cell membranes. Fluorescence microscopy using MitoTracker and an antibody against caveolin-1, a major structural protein of caveolae, showed that the caveolae were concentrated at specific areas of the edge of the cell where mitochondria were located in close proximity (Fig. 4*A*). Treatment of cells with siRNA of caveolin-1 completely abolished the expression of caveolin-1, indicating destruction of the caveolae, but had no significant effect on the morphology and distribution of the mitochondria. Because caveolin-1 knockdown with its siRNA significantly inhibited shear stress-induced ATP release (Fig. 3*B*), we investigated whether caveolae or caveolin-1 could be implicated in shear stress-induced mitochondrial ATP generation. Shear stress-induced mitochondrial ATP generation was not markedly observed in cells transfected with caveolin-1 siRNA, in clear contrast with control cells transfected with caveolin-1 scrambled siRNA (Fig. 4*B*). These findings indicated that caveola or caveolin-1 had a critical role in shear stress-induced mitochondrial ATP generation.

#### Mitochondrial ATP generation triggered shear stress-mediated Ca^2+^ signaling.

ECs were subjected to shear stress, and changes in [Ca^2+^]_i_ were monitored using the Ca^2+^-sensitive dye Fluo-4. Shear stress evoked a rapid increase in [Ca^2+^]_i_ in cells treated with caveolin-1 scrambled siRNA; when shear stress was terminated, [Ca^2+^]_i_ returned to its basal level (Fig. 4*C*). This Ca^2+^ response to shear stress did not occur in cells treated with caveolin-1 siRNA. Blockage of mitochondrial ATP generation with oligomycin, CCCP, or rotenone abolished the shear stress-mediated Ca^2+^ response. The Ca^2+^ response was blocked by EGTA but not by MitoTEMPOL, indicating that the Ca^2+^ response is due to the influx of extracellular Ca^2+^ into cells and that ROS produced by mitochondria do not play a part in Ca^2+^ responses. These findings indicate the following order of events: mitochondrial ATP generation occurred in response to shear stress followed by release of ATP, which induced an increase in [Ca^2+^]_i_ via the influx of extracellular Ca^2+^.

## DISCUSSION

We used the mitochondria-targeting ATP biosensor mitAT1.03 for real-time imaging of mitochondrial ATP generation in cultured ECs under various flow conditions. ECs rapidly responded to flow with a marked increase in the generation of ATP by mitochondria. The mitochondrial response was reversible and dependent on the intensity of shear stress. In recent years, it has been demonstrated that shear stress affects the morphology and functions of endothelial mitochondria (30). For example, when human umbilical vein ECs were exposed to shear stress for 36 h, mitochondrial biogenesis increased according to shear stress intensity, resulting in an increase in the number of mitochondria in the cell (23). Exposure of porcine aortic ECs to shear stress for 48 h increased their mitochondrial membrane potential and intracellular ATP levels (24). In bovine aortic ECs, shear stress first induced a dynamic change in the morphology of the mitochondria, including fragmentation and fission, followed by a decrease in the respiration rate and an increase in the generation of ROS (9). On the other hand, there is also a report of exposure to shear stress not causing any significant morphological changes in EC mitochondria (18). Furthermore, shear stress increases [Ca^2+^]_i_ in ECs, which leads to nitric oxide production; these responses have been shown to secondarily modulate the mitochondrial bioenergetics and ROS production in the cells (21, 33). All of these findings represent relatively long-term effects of shear stress on the mitochondria. In contrast, the present study demonstrated the real-time effect of shear stress on mitochondrial ATP generation, which is, to the best of our knowledge, the earliest mitochondrial response to shear stress exposure.

ECs release ATP in response to shear stress; in turn, the released ATP activates P2X4 purinoceptors to cause cellular influx of extracellular Ca^2+^ (6, 38, 39). However, how ATP release is evoked by shear stress remains unknown. In the present study, we showed that shear stress caused the augmentation of mitochondrial ATP generation and that blockade of mitochondrial ATP generation with inhibitors of mitochondrial oxidative phosphorylation abolished shear stress-induced ATP release. Treatment of ECs with 2-DG, an inhibitor of cytosolic glycolysis, had no effect on either shear stress-induced ATP generation or ATP release. These findings clearly indicated that the ATP released by shear stress originated from mitochondria. Until recently, it has been assumed that ECs obtain most of their energy for their activities, including cell movement, cytoskeleton contraction, active transport of substances, and ion channel opening, from glycolysis and that the primary functions of the mitochondria are the production of ROS and mediation of redox signaling (27). This study is the first to demonstrate a novel role of mitochondria as mechanosignaling organelles that are able to transduce shear stress into ATP generation, triggering ATP release and purinoceptor-mediated Ca^2+^ signaling that lead to biological responses.

**Fig. 5.**
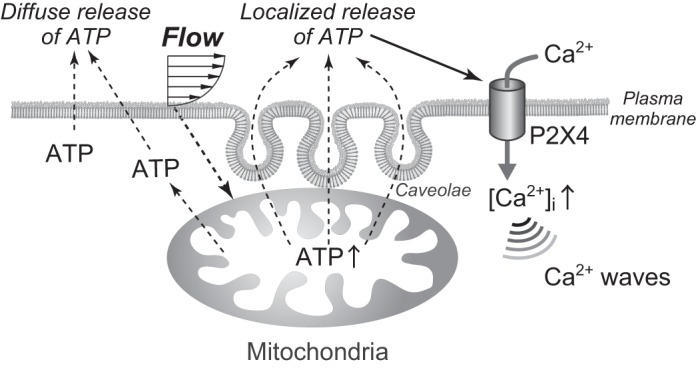
Schematic diagram of the proposed caveolae-associated purinergic Ca^2+^ signaling in endothelial cells (ECs) exposed to shear stress. Shear stress induces both diffuse ATP release from the entire surface of the cell membrane and a localized ATP release from caveolae-rich cell regions. Since the localized ATP release reaches to more than 10 μM, it can activate the ATP-operated cation channel P2X4, thereby causing an influx of extracellular Ca^2+^ into cells. The increase in the intracellular Ca^2+^ concentration evokes a Ca^2+^ wave that starts at the caveolae and propagates throughout the entire cell. Mitochondria increase their ATP generation in response to shear stress exposure of the ECs and play a role as the source of the ATP released by ECs. The mechanism underlying the induction of increased mitochondrial ATP generation in response to shear stress in ECs remains unknown.

In previous studies, we demonstrated that shear stress induced highly concentrated, localized ATP release in the caveolae-rich regions of EC plasma membranes (37). Disruption of caveolae using caveolin-1 siRNA or methyl-β-cyclodextrin, which depletes membrane cholesterol, was found to abolish localized ATP release, indicating that intact caveolae are a prerequisite for this release. Recently, it has become apparent that there are physical and functional interactions between caveolae and mitochondria (28). Mitochondria have been observed in close contact with the plasma membrane in several mammalian cell types (19), including ECs, HeLa cells (17), hepatocytes (15), and cardiomyocytes (12, 34). For example, in HeLa cells, up to 10% of the total plasma membrane area is covered with mitochondria (known as subplasmalemmal mitochondria). Electron microscopy has demonstrated close apposition between mitochondria and caveolae in cardiomyocytes and that direct connections form between these organelles and membrane microdomains during ischemic stress (16). It is thought that mitochondria located in close proximity to caveolae supply them with highly concentrated ATP. There may be unidentified structures that link mitochondria to caveolae through which such ATP transfer can occur. Figure 5 shows a schematic diagram of the proposed caveolae-associated purinergic Ca^2+^ signaling in ECs exposed to shear stress.

Caveolins are localized in mitochondria as well as in the caveolae (16, 25). They can transfer between the caveolae and mitochondria, which plays an important role in adaptation to cellular stress and injury. Caveolin-1 deficiency in mice has been shown to impair mitochondrial functions; it causes an accumulation of free cholesterol in mitochondrial membranes, which increases membrane condensation and reduces the efficiency of the respiratory chain and intrinsic antioxidant defense, thereby increasing the cellular susceptibility to apoptosis (8). Loss of caveolin-1 also induces an increase in mitochondrial ROS and intracellular H_2_O_2_ production in ECs (31). In the present study, we showed that knockdown of caveolin-1 with its siRNA abolished shear stress-induced mitochondrial ATP generation. Although the functions of caveolin-1 localized in the mitochondria have not yet been clarified, it seems that caveolae or caveolins play a critical role in shear stress-induced mitochondrial ATP generation.

ECs are known to respond to shear stress and increase the production of ROS in mitochondria as well as the Ca^2+^ concentration in both the cytoplasm and mitochondria. These increased ROS and Ca^2+^ affect mitochondrial ATP generation by modulating the activity of the electron transport chain and ATP synthase, whereas the produced ATP influences ROS production and Ca^2+^ concentration. These mutual interplays among ROS, Ca^2+^, and ATP are thought to play an important role in maintaining EC homeostasis (1, 10). In the present study, we examined whether Ca^2+^ and ROS are involved in shear stress-induced mitochondrial ATP production and ATP release. Neither addition of EGTA to culture media, which removes extracellular Ca^2+^ and abolishes the shear stress-induced Ca^2+^ influx, nor treatment of ECs with MitoTEMPOL, which inactivates mitochondrial ROS, exerted any influence on shear stress-induced ATP generation and ATP release. These findings indicate that Ca^2+^ influx and ROS do not play a part in these events. On the other hand, not only the influx of extracellular Ca^2+^ but also Ca^2+^ release from the endoplasmic reticulum (ER) also occurs in response to shear stress, and mitochondria play an important role through Ca^2+^ uptake/release of the Ca^2+^ released by the ER (29). However, it remains unclear whether the Ca^2+^ released from the ER is related to the shear stress-induced ATP production/ATP release.

At present, it remains unclear how shear stress causes augmented mitochondrial ATP generation in ECs. Although as yet there is no direct evidence, several possible molecular mechanisms can be speculated. The first relates to the cytosolic pH level. Studies in rat and bovine aortic ECs have shown that cytosolic pH decreased within seconds of exposure to shear stress (26, 42, 43). This decrease appeared to be mediated by alterations in the functions of membrane ion transporters, such as ${\text{HCO}}_{3}^{-}$/Cl^−^ and Na^+^/H^+^ exchangers. The decrease in pH (i.e., the increase in H^+^ concentration) may, in turn, affect the activity of ATP synthase. Another possible mechanism relates to the O_2_ concentration. In a recent study, we (36) demonstrated that exposure of ECs to shear stress resulted in a reduction in the cholesterol content of their plasma membranes. Cholesterol acts as a barrier that limits the permeability of the lipid bilayer membrane of cells to O_2_ (14, 22, 32); thus, reduction of the membrane cholesterol content increases the mitochondrial O_2_ concentration, which may influence electron transfer chain reactions. A further possible mechanism involves the cytoskeleton. Mitochondria anchor to the cytoskeleton via actin-binding complexes in the outer membrane (7). Shear stress applied to the plasma membrane may be transmitted to the mitochondria via the cytoskeleton, affecting mitochondrial ATP generation. However, the biophysical process underlying the transduction of cytoskeletal strain to changes in mitochondrial functions remains unknown. It is possible that other factors may be implicated in the effects of shear stress on mitochondrial ATP generation, and further studies are needed to precisely elucidate the underlying mechanism.

## GRANTS

This work was supported by Scientific Research from the Japan Agency for Medical Research and Development under Grant JP18gm0810006.

## DISCLOSURES

No conflicts of interest, financial or otherwise, are declared by the authors.

## AUTHOR CONTRIBUTIONS

K.Y. and J.A. conceived and designed research; K.Y. and J.A. performed experiments; K.Y., H.I., and J.A. analyzed data; K.Y., H.I., and J.A. interpreted results of experiments; K.Y. and J.A. prepared figures; K.Y. and J.A. drafted manuscript; K.Y. and J.A. edited and revised manuscript; K.Y., H.I., and J.A. approved final version of manuscript.
